# Influence of inter- and intramolecular H-bonding on the mesomorphic and photoswitching behaviour of (*E*)-4-((4-(hexyloxy)phenyl)diazenyl)-*N*-phenyl benzamides†

**DOI:** 10.1039/d0ra03024d

**Published:** 2020-05-27

**Authors:** B. N. Sunil, Paresh Kumar Behera, Ammathnadu S. Achalkumar, G. Shanker, Gurumurthy Hegde

**Affiliations:** Center for Nano-materials and Displays, BMS R and D Centre, B.M.S. College of Engineering Bangalore 560019 India murthyhegde@gmail.com; Department of Chemistry, B.M.S. College of Engineering Bangalore 560019 India; Department of Chemistry, Indian Institute of Technology Guwahati Guwahati 781039 Assam India; Department of Chemistry, Jnana Bharathi Campus, Bangalore University Bangalore 560056 India

## Abstract

We report on the synthesis, phase behaviour and photoswitching studies of new azo linked rod-shaped molecules. These novel materials consist of three phenyl rings separated by a diazo, amide linkage with a hexyloxy tail and 2,4-substituents at either end of the phenyl ring. The mesomorphic behaviours, phase transition temperature including the enthalpies were characterized by polarizing optical microscope (POM) and differential scanning calorimetry (DSC). The influence of inter- and intramolecular hydrogen bonding on mesomorphic and photoisomerization was studied. Photoisomerization studies carried out both in the solid and liquid phase show the quick *E*–*Z* transition with prolonged thermal back relaxation (*Z*–*E*) by using UV-Visible spectroscopy. This interesting behaviour could be attributed to the presence of the hexyloxy tail, lateral electron withdrawing group and the influence of inter- or intramolecular hydrogen bonding. Excellent bright and dark states were accomplished using one of these materials in optical storage device. Further tuning is necessary to employ them for real applications.

## Abstract 2

We report on the synthesis, phase behaviour and photoswitching studies of new azo linked rod-shaped molecules.

## Graphical abstract

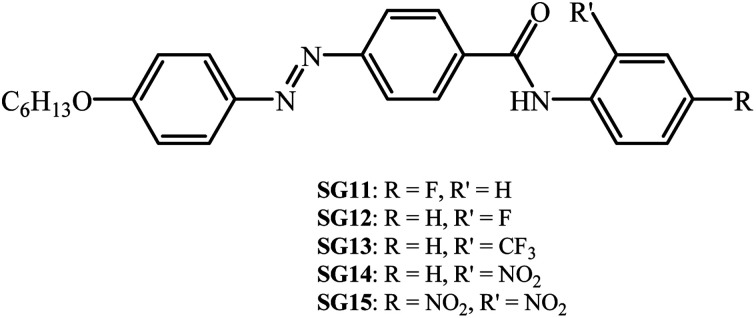

## Introduction

Aromatic azobenzene compounds are most promising candidates for optical switching applications due to their photosensitivity.^1^ Azobenzene and its derivatives are well known as stimuli responsive materials,^2^ due to their –N=N– double bond which responds to light. Therefore, irradiation with the appropriate wavelength leads to reversible *trans*–*cis* isomerization.^3^ This process assists in the major structural change and leads to a difference in the change of the polarity of the molecule.^4^ Upon UV irradiation, which corresponds to the π–π* transition, there is a transformation from the thermodynamically most stable rod-like molecular form of *trans* isomer (*E*) into a meta-stable bent, *cis* isomer (*Z*). The azo chromophore exhibits a two-absorption peak, high intensity π–π* absorption peak in the UV region and lower intensity n–π* absorption peak in the visible region.^5^ The reverse isomerization can be induced in two ways, one is by shining the visible light (*λ* ∼ 450 nm) whose wavelength corresponds to n–π* transition. Another is through the reverse mechanism that will undergo in the “dark” by a process known as thermal back relaxation.^6^ These special optical properties make azobenzene derivatives as a class of much sought after photoresponsive moieties for the exploration in advanced technologies.^7–10^

Understanding the mechanism of the *trans*–*cis*–*-trans* photoisomerization is the need to improve the applications of these materials for photonic application. Azobenzene derivatives are one among the photochromic materials, which are most attractive in the field of optical storage devices due to their compatibility with the mesogens to induce rich polymorphism, higher thermal stability and their photoisomerization properties.^11–13^ However, tailoring of these compounds with high chemical (without any degradation) and thermal stability (over a broad range of temperature), along with liquid crystalline properties, with stable *cis* isomer still remains as a challenge for researchers who are working in this area. Moreover, the thermal *cis*–*trans* isomerization of azo derivatives makes them excellent candidates for optical storage devices due to high stability in the *cis* form and the reversibility of the isomerization.^14,15^ In addition, azobenzene derivatives have been proposed for potential applications in the areas of nonlinear optics,^16^ chemo sensors,^17^ liquid crystals,^18^ photochemical molecular switches,^19^ molecular shuttles^20^ and drug delivery.^21^

Recently, we have designed and studied the optical storage properties of several azobenzene derivatives, namely, the azobenzene dimers with aromatic and aliphatic spacers,^22^ fluorinated azobenzene esters,^23^ photopolymerizable azobenzene derivatives,^24^ bent-shaped azobenzene monomers,^25,26^ alkoxy azobenzene derivatives with coumarin moieties,^27^ siloxane based azobenzene derivatives,^28^ hydrophilic/hydrophobic-based azobenzene mesogens^29^ and amide functionalized azobenzene derivatives with intermolecular H-bonding.^30^ The presence of an electronegative moiety in the case of amide containing azobenzene units leads to another possibility of a weak hydrogen bonding due to the lone pair of electrons present on electronegative atom.^31^ This can be inter- or intramolecular-bonding depending on the position of the electronegative moiety on the amide containing azobenzene molecule. Nevertheless, the presence of H-bonding restricts the free movement of azo molecule. Considering all these aspects, we have designed a new series of molecules with electron withdrawing group substituted at *ortho* and or *para* position to amide linkage.

**Fig. 1:**
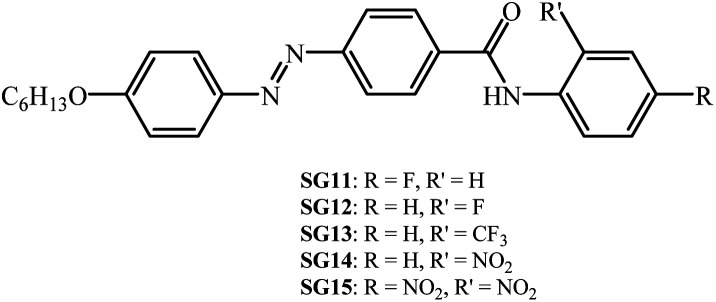
Synthesized chemical structures of the alkoxy azobenzene derivatives with different electron withdrawing groups at lateral/terminal position of phenyl ring to the amide moiety.

In particular, we have synthesized (*E*)-4-((4-(hexyloxy)phenyl)diazenyl)-*N*-phenyl benzamides with various electron withdrawing groups (EWG) at *ortho*/*para* position to the amide linkage (Fig. 1). The molecular structural characterization with standard analytical techniques with FT-IR and ^1^H NMR, their thermal behaviour was characterized with polarized optical microscopy (POM) and differential scanning calorimetry (DSC). The thermal behavior of the materials are summarized in Table 1. Further their photoisomerization behaviour was monitored with UV-Vis spectroscopy. The –F, –CF_3_ and –NO_2_ groups are attached to *ortho*/*para* positions of the phenyl terminal ring to the amide moiety. The effect of polarity, position and H-bonding on the mesomorphism and photoisomerization behaviour of synthesized compounds was investigated. The kinetics of thermal reverse isomerization was investigated by UV-Vis spectroscopy.

**Table 1:** Phase transition temperatures, enthalpy of transition (Δ*H*, kJ mol^−1^) for all the compounds obtained from DSC thermograms upon cooling cycles at 5 °C min^−1^^a^

Compound code	Phase transition temperature (°C), (Δ*H*, kJ mol^−1^)
SG11	Iso 209.6 (0.78) N 190.5 (8.59) Cr
SG12	Iso 155.72 (2.5) N 152.8 (6.5) Cr
SG13	Iso 186.30 (27.9) Cr
SG14	Iso 209.73 (0.93) N 193 (33.04) Cr
SG15	Iso 175.16 (1.17) N 171.02 (29) Cr 156.9 (3.8) Cr′

a Abbreviations: Cr = crystalline, Cr′ = crystal to crystal, N = nematic phase, Iso = isotropic liquid. Values in bracket corresponds to enthalpy of transition (Δ*H*, kJ mol^−1^).

## Results and discussion

**Scheme 1:**
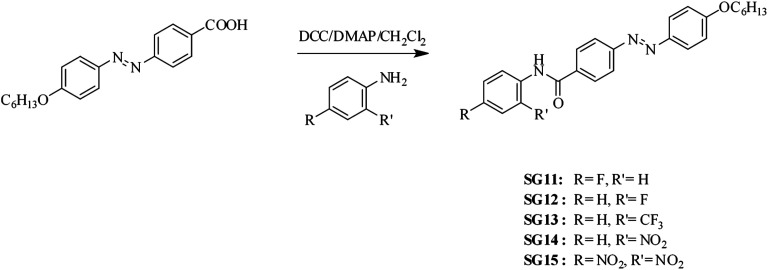
Synthetic route followed to synthesize the (*E*)-4-[(4-hexyloxyphenyl)diazenyl]-*N*-phenyl benzamides.

Synthetic route of (*E*)-4-[(4-hexyloxyphenyl)diazenyl]-*N*-phenyl benzamides bearing electron withdrawing groups at the *para*/*ortho* position to the amide linkage as depicted in Scheme 1. The azobenzene based benzamides were prepared by coupling the (*E*)-4-[4-(4-hexyloxy)phenylazo]benzoic acid with substituted aniline derivatives in the presence of 1,3-dicyclohexylcarbodiimide (DCC), 4-(*N*,*N*-dimethyl amino)pyridine (DMAP) as a coupling agent. The crude product was purified by column chromatography using silica gel (60-120 mesh), followed by recrystallization from methanol. The detailed synthetic procedure for the intermediate (*E*)-4-[4-(4-hexyloxy)phenylazo]benzoic acid was reported in our previous paper.^30^ The synthetic procedure and characterization data obtained from FT-IR, ^1^H NMR for final compounds are compiled in Experimental section.

### Mesomorphic behaviour

Phase transition temperatures and their associated enthalpies of all the compounds are summarised in Table 1. All investigated compounds are liquid crystalline in nature except compound SG13. From Table 1, it is seen that the compound SG11 having intermolecular hydrogen bonding shows higher transition temperature as compared to SG12, which is having intramolecular H-bonding. The compound SG12 exhibits very short range of nematic phase around 3 °C. The mesophase stability depends on intermolecular H-bonding between the molecules for the mesomorphic compound, in which the polarity of the molecules plays an important role. The nature of the substituent influences the extent of conjugation, which alters the resultant dipole moment and polarizability of the molecule.^32–36^

The nematic phase between SG12 and SG15 is almost 3 degrees this could be attributed to the EWG in the lateral position (F) in the case of SG12, and lateral and terminal position (NO_2_) in SG15 but the melting and clearing temperatures are different. SG14 has one NO_2_ laterally attached and exhibit an increased mesophase range. At present we are not in a position to pinpoint the structure–property relation in terms of mesophase range, melting and clearing points. The observed changes are due to the summation of different noncovalent interactions, functional groups, hydrogen bond (inter and intra), extent of conjugation, resultant dipole moment and overall shape of the compounds.^32–36^

**Fig. 2:**
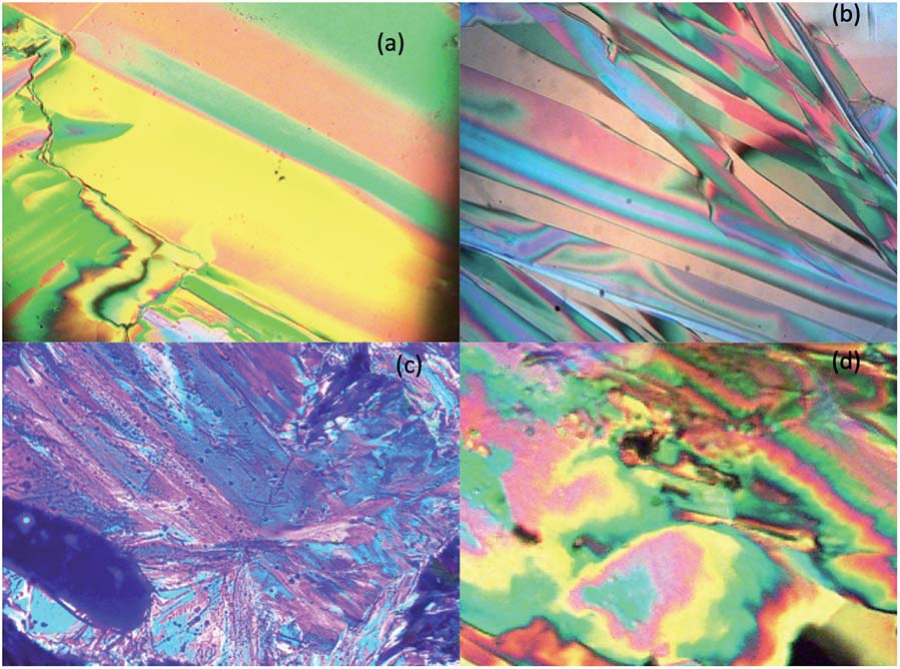
Observed POM texture for SG11 compound upon cooling from isotropic liquid; (a) nematic phase at 198 °C, (b) nematic to crystalline transition at 190.5 °C, (c) crystalline phase at 188 °C and (d) nematic phase observed for the compound SG15 at 174 °C (magnification is 10× and 200 μm).

**Fig. 3:**
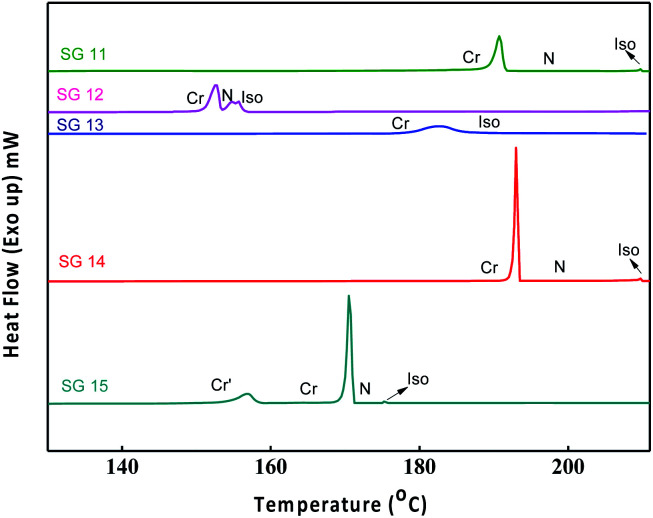
DSC thermograms recorded upon cooling cycle at the rate of 5 °C min^−1^ for all the compounds.

The optical textures were captured upon cooling from isotropic liquid at a cooling rate 2 °C min^−1^ (Fig. 2). Fig. 2a and b shows the nematic phase and nematic to crystalline phase transition which was captured during cooling from isotropic liquid for compound SG11. Further cooling leads to crystalline phase (Fig. 2c). The nematic phase was observed in SG15 as shown in Fig. 2b. In case of compounds SG12, it was found to be difficult to capture nematic phase due to the narrow thermal range. Fig. 3 indicates the DSC thermogram for all the final compounds which are recorded upon cooling cycle at the rate 5 °C min^−1^.

### Photoswitching studies in solution

**Fig. 4:**
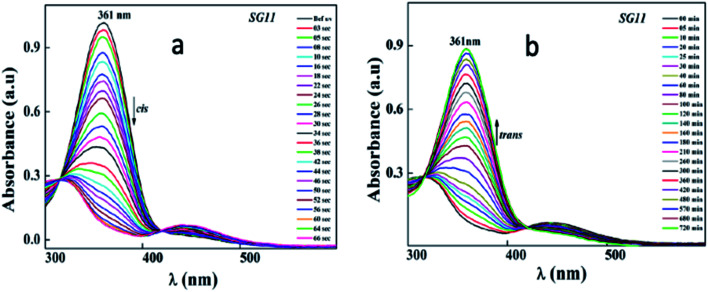
Representative time dependent absorption spectra for compound SG11, (a) corresponds to *E*–*Z* isomerization during UV illumination and (b) corresponds to *Z*–*E* isomerization during thermal back relaxation.

**Fig. 5:**
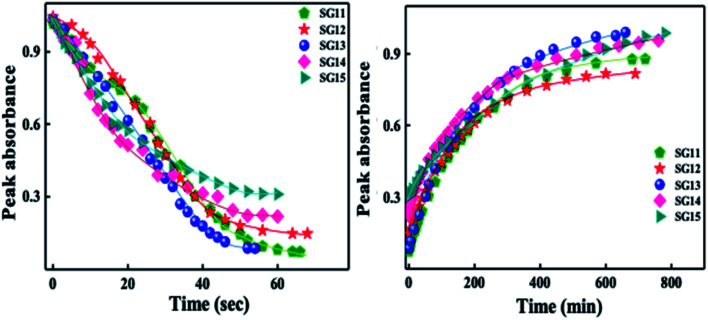
Peak absorbance plot *versus* different time intervals, which is plotted by extracting the absorbance values at their peak wavelength from absorption spectra of all compounds: left side of the image corresponds to UV illumination (from absorption spectra of Fig. 4a and S1†) and right side of the image corresponds to thermal back relaxation (from absorption spectra of Fig. 4b and S2†).

Identical absorption spectra were observed for all the compounds due to their similar molecular structure. The only change in structure is the position of electron withdrawing group on phenyl ring to the amide linkage. The photoisomerization experiments were conducted in the chloroform solution of these compounds (concentration: 1.0 × 10^−5^ mol L^−1^, optical path length: 1 cm). The observed results of the UV-Visible absorption spectra of compound SG11 is given in a Fig. 4. Fig. 4a and b corresponds to time dependent absorption spectra obtained during UV illumination and thermal back relaxation, respectively. As a result of UV illumination, absorption band at UV region (*λ* ∼ 360 nm) corresponds to a high intensity π–π* transition gradually decreases; whereas, band at visible region (*λ* ∼ 450 nm) corresponds to low intensity n–π* transition simultaneously increases. The change in absorption spectra was recorded until *E* or *trans* isomer reaches its photostationary state (PSS). The compound SG11 took more time (∼60 s) to reach its PSS as compared with other compounds. Intermolecular H-bonding between the molecules restrict a free rotation, resulting in an increased photosaturation time of *E*–*Z* isomerization. Fig. 5a shows peak absorbance *versus* time plot of all compounds, which is plotted by extracting absorbance values at their peak wavelength from absorption spectra (Fig. 5a and S1†) of respective compounds.

After reaching PSS, there was no significant changes in the absorption spectra, which corresponds to the *trans*–*cis* isomerization. It suggests that, the ratio of *trans* and *cis* isomers remains unchanged after ∼60 s of UV illumination. For *trans*–*cis* isomerisation, photoconversion efficiency (CE) was determined by using eqn (1) at PSS.^26^1
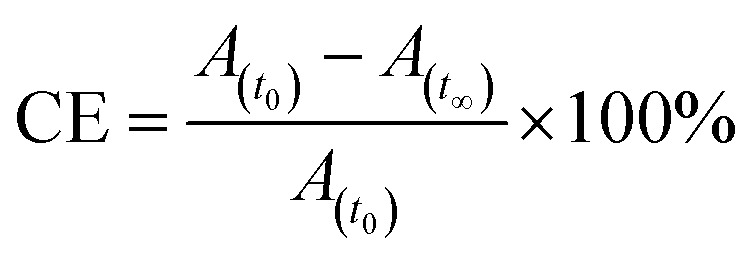
where, CE is photoconversion efficiency, *A*_(*t*_0_)_ and *A*_(*t*_∞_)_ is absorbance before and after UV illumination respectively.

The photoconversion efficiency is very important for expressing the photoresponsive behaviour of azo derivatives. The calculated photoconversion efficiency of *trans* isomer at PSS of these compounds was shown in Table 2. In case of SG11, the extent of isomerization was found to be ∼92%; while the lowest CE was noted in the compound SG15 (∼69%). This indicates that the CE decreases, when the azo derivatives are substituted with the groups of increased electron withdrawing character.

**Table 2:** Summarized data of photosaturation time, *Z*–*E* conversion time and photoconversion efficiency (CE)

Compound code	Photosaturation time (s)	*Z*–*E* conversion (h)	CE (%)
SG11	60	11.33	91.92
SG12	54	9.0	85.82
SG13	50	9.5	91.48
SG14	48	9.66	76.23
SG15	50	13.0	69.58

Generally, reverse isomerization can be brought by two ways: one is by the irradiation of white light at higher wavelength and other one is by keeping the solution in the dark, which is called as thermal back relaxation. In this study, reverse isomerization was determined by keeping a sample in the dark after PSS was reached. The changes in the absorption spectra have monitored at successive time intervals during the thermal back relaxation. The compound SG15 exhibited longest thermal back-relaxation time (∼13 h); while the shortest duration was recorded for SG12 (∼9 h). The time dependent absorption spectra of compounds during thermal back relaxation are as shown in the Fig. 5b, which was extracted from Fig. 4b and S2.† The peak absorbance was plotted based on the absorbance at ∼361 nm with different intervals of time. The difference in thermal back relaxation time is most likely due to the position at which the EWGs are substituted. This in turn affects the extent of influence by EWG on inter- and intramolecular-bonding and result on the thermal back relaxation of *cis* isomer to a greater extent.

### Kinetic studies

**Fig. 6:**
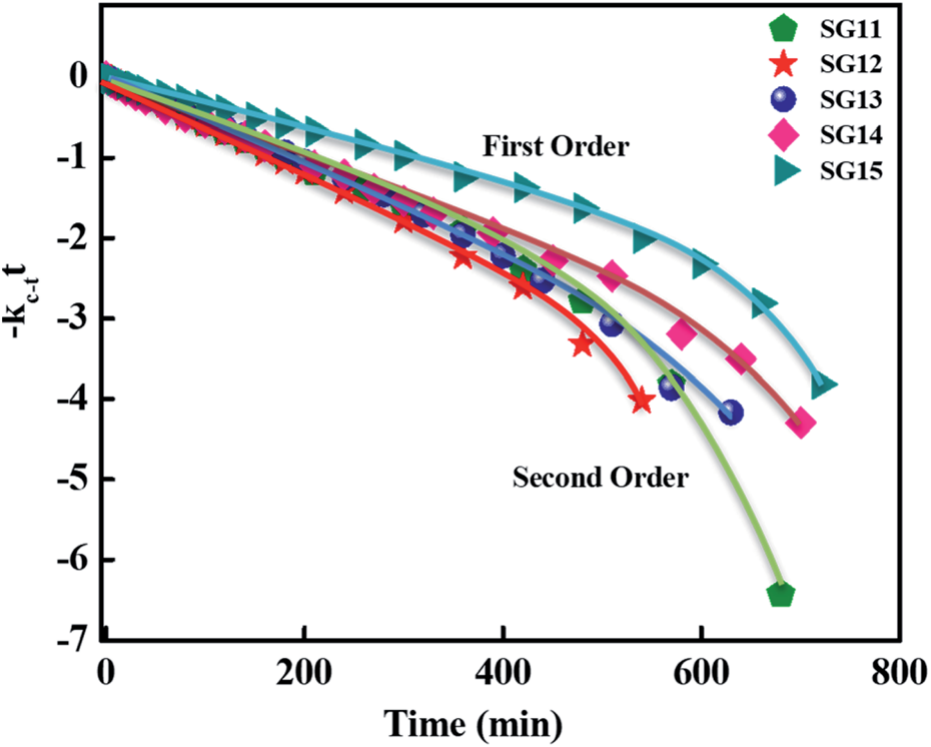
First-order plot as a function of time for the *cis*–*trans* thermal isomerization for compounds such as SG11, SG12, SG13, SG14 and SG15 are evaluated at room temperature.

For reversible thermal *cis*–*trans* isomerization, first-order kinetics of the reaction was obligatory to study. The unimolecular reversible isomerization reaction obeys eqn (2) and Fig. 6 shows the first-order plot which was evaluated by fitting the experimental data to the eqn (2) of thermal *cis*–*trans* isomerization.^29^ At room temperature (∼27 ± 1 °C), experiment was monitored using UV-Vis spectroscopy.2
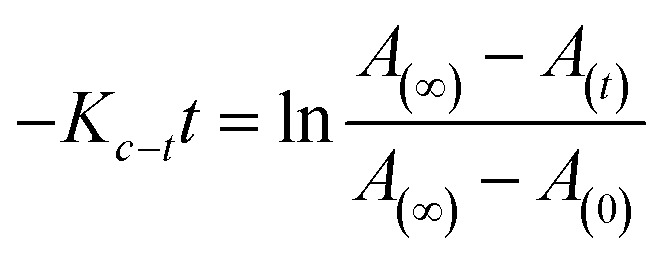
where, *A*_(*t*)_, *A*_(0)_ & *A*_(∞)_ denotes the absorbance of peak wavelength at time *t*, time zero & infinite time, respectively, *t* is the relaxation time of the corresponding *cis* isomer. From Fig. 6, reaction was first order at a certain time of interval, there after deviated to second order from first order. This deviation of reaction might be due to long thermal back relaxation and it was affected by the experimental temperature conditions,^29^ since the reverse isomerization has been carried out in solution. Here, one can notice the predominant changes in the thermal back relaxation time with respect to the molecular structure of azo chromophore.

### Reason behind this phenomena

We speculated the possible reason behind this phenomena might be the presence of intermolecular H-bonding between the molecules and intramolecular H-bonding within the molecules. Another possibility is the electronic effects of different functional groups, which also influences the H-bonding to a significant extent. FT-IR is a powerful tool to investigate the inter- and intramolecular H-bonding;^37^ in the compounds having intermolecular hydrogen bonding, a shift in the wavenumber occurs with changing the concentration; whereas, the wavenumber range remains constant in case of intramolecular hydrogen bonding. The recorded IR spectra of representative compounds SG11 and SG14 with different concentration are shown in Fig. S3† and chloroform is used as a solvent.

**Fig. 7:**
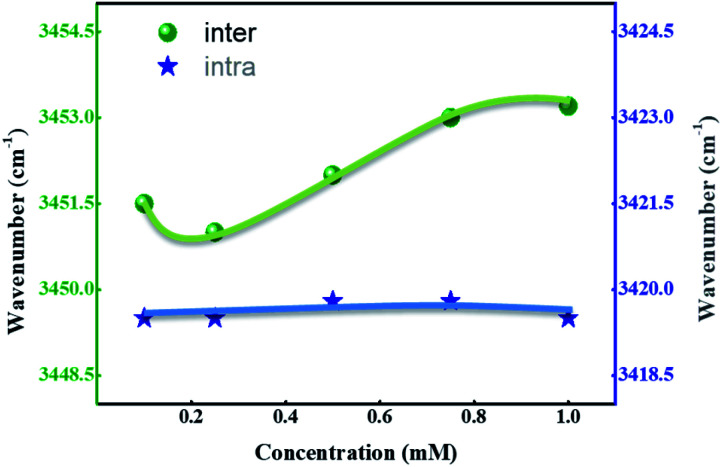
Wavenumber *versus* concentration corresponds to inter- and intramolecular N–H stretching.

Fig. 7 represents a wavenumber *versus* concentration graph, shows the maximum absorption band of SG11 corresponds to intermolecular H-bonding, which is shifted to higher wavenumber with concentration increases. The changes in spectral behavior is due to presence of intermolecular H-bonding between the molecules. In the case of SG14, the maximum of the N–H stretching band remains same wavenumber with the concentration changes. The absorption band unaltered with the increased concentration, which is characteristic for intramolecular H-bonding. Intramolecular hydrogen bonding is formed between the N–H in amide group and the oxygen in nitro group at *ortho* position of phenyl ring.^38^ Therefore, FT-IR spectra confirms the existence of mentioned inter- and intramolecular H-bonding in respective compounds.

Table 3 summarizes the stretching frequency values which correspond to the maxima of the N–H stretching band for the compounds SG11 with the intermolecular H-bonding and SG14 with the intramolecular H-bonding, respectively. The significant changes in N–H stretching band around 3452.5 cm^−1^ corresponds to intermolecular H-bonding as the concentration varies; whereas, stretching frequency corresponding to intramolecular hydrogen bonding behavior relatively remains constant. The N–H stretching frequency corresponds to N–H group was shifted to around 3419.5 cm^−1^ compare to SG11. The difference in wave number is 33 cm^−1^, which is in agreement with intramolecular H-bonding behavior of SG14.

**Table 3:** Summarized data corresponds to maxima of the N–H stretching absorption bands with respect to concentration

Concentration (mM)	Wavenumber (cm^−1^)	
	SG11 (inter)	SG14 (intra)
0.1	3451.5	3419
0.25	3451.0	3419
0.5	3452.0	3420
0.75	3453.0	3420
1	3453.2	3419

**Fig. 8:**
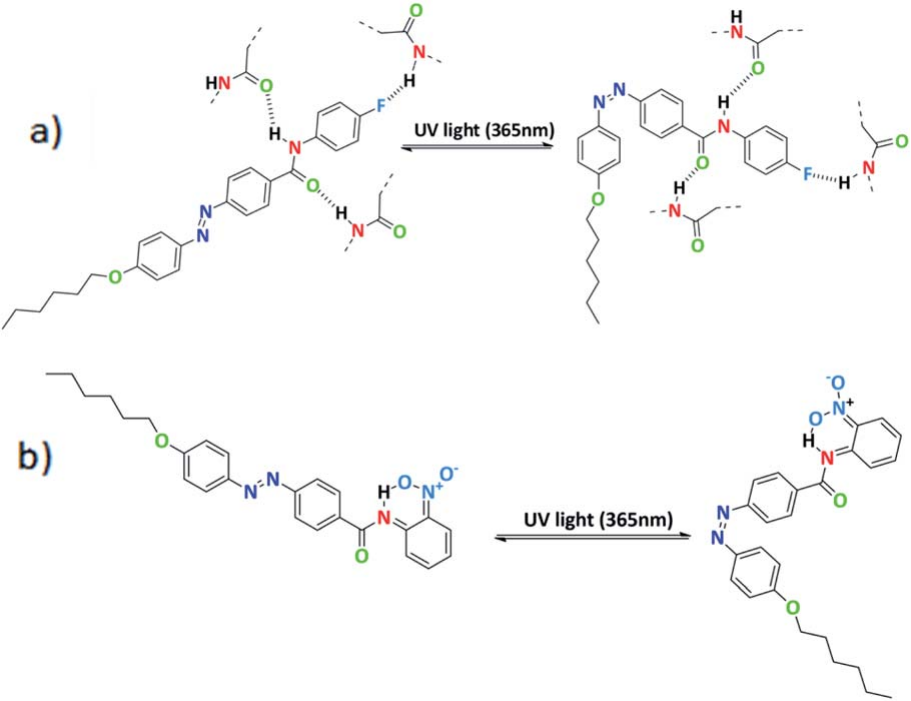
(a) Schematic representation of *para* substituted azo molecules with intermolecular H-bonding and (b) *ortho*-substituted azo molecules with intramolecular H-bonding.

Further, schematic representation of stable intermolecular H-bonding in SG11 is shown in Fig. 8a. There is a possibility of stable H-bonding formation between fluoro and hydrogen atom of amide linkage or oxygen and hydrogen atom of amide group. The negative inductive effect (−*I*) of fluoro group influences the H-bonding between molecules to a significant extent.^39^ Further, the extent of H-bonding on fluoro group and amide linkage influences the life time of *cis* isomer at PSS. This leads to a retardation of free molecular movement in this compound to a greater extent and increases the time required to revert to its original state from PSS.

In case of SG14, there is an existance of intramolecular H-bonding between the oxygen atom of nitro group at *ortho* position and the hydrogen atom of amide linkage (Fig. 8b).

The shorter life time of *cis* isomer at PSS, due to six membered transition state in the system creates a non-planarity and result on the macroscopic structure.^40^ This leads to the fast thermal back relaxation and similar results was observed in case of SG12 and SG13. But, interestingly SG15 shows longest thermal back relaxation time which is having EWG at both *ortho* and *para* position. It should be noted that the nitro group in the *para* position contributes toward the intermolecular H-bonding as well in addition to intramolecular H-bonding. In addition, electronic effect of the substituted group influences on the inter- and intramolecular H-bonding and it is crucial for tuning the optical storage device with respect to molecular structure.

### Photoswitching studies in solid cell

**Fig. 9:**
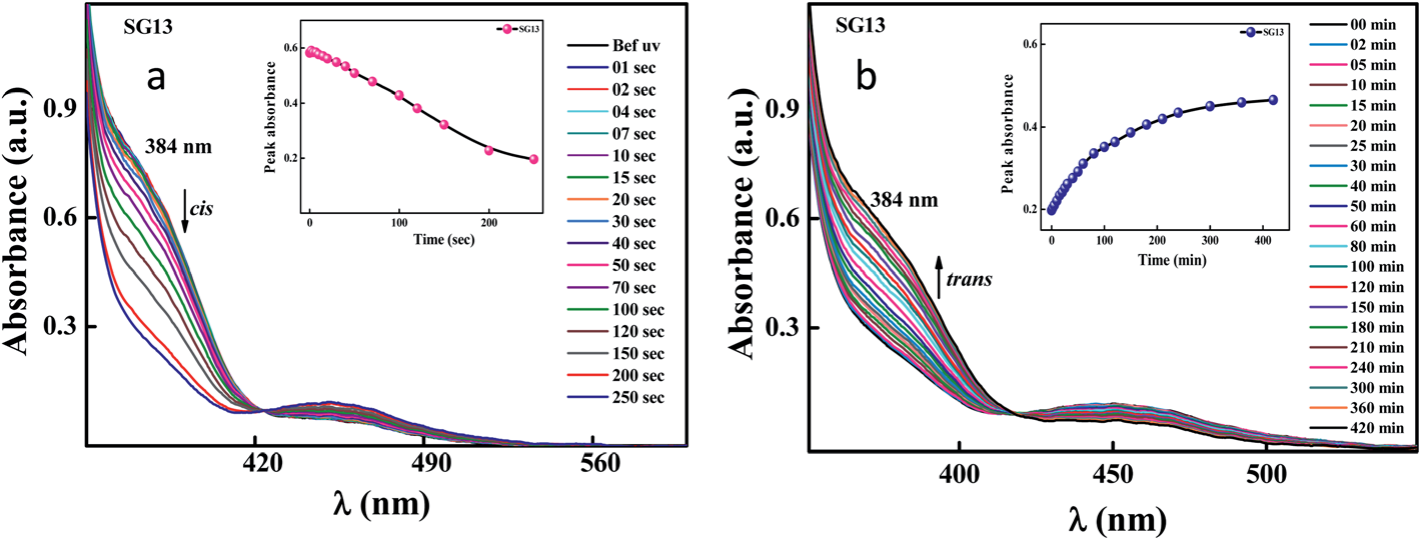
Spectral investigation of LC cell during UV illumination and thermal back relaxation for compound SG13, respectively. Photostationary state of *trans* isomer was achieved around 200 s (graph a); whereas, reverse thermal isomerization takes around 6 h (graph b). Intensity of the illuminated UV light was 1 mW cm^−2^.

To see the potential ability of the above said materials, the solid cell was fabricated by using two ITO glass substrate coated with polyimide coated (planar geometry with antiparallel rubbing) with 5 μm thickness using glass spacers. Guest–host mixture was filled by using capillary action and mixture is prepared physically using SG13 (non-liquid crystalline in nature) which act as a guest and liquid crystal E7 act like a host material. Fig. 9 shows the spectral data of the reversible *trans*–*cis*–*trans* isomerisation for the solid cell. The liquid crystal cell achieved its photostationary state at around 200 s (Fig. 9a); whereas, the reverse thermal back relaxation was observed at around 6 h (Fig. 9b). The peak absorbance plots at different interval of times, which was plotted by extracting absorbance values at their peak wavelength from the absorption spectra of UV illumination (Fig. 9a) and thermal back relaxation (Fig. 9b), respectively.

### Optical storage device

**Fig. 10:**
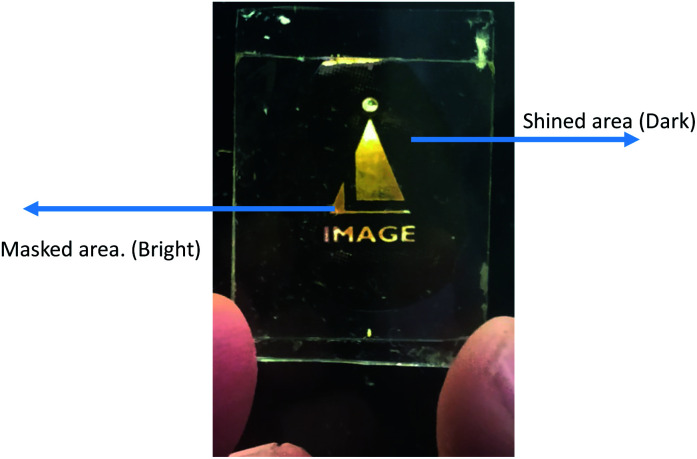
Fabricated optical storage device using guest–host mixture of compound SG13 (as a guest) and E7 liquid crystal (as a host). Bright state corresponds to masked area and dark state corresponds to exposed/shined area to UV light (5 mW cm^−2^). Mask is taken with the permission from Image Labels Pvt Ltd, Bengaluru.

To prepare the device, the same cell was used for study the guest–host composite mixtures explained in previous section. Suitable mask was kept above the said cell and UV light was shined on the sample. The wavelength of shined light was 365 nm and intensity was 5 mW cm^−2^. During illumination of UV light, materials transform from one state (ordered state) to another state (disordered state). Fig. 10 shows excellent bright and dark states under the crossed polarizers where bright state corresponds to masked area (*i.e.* molecules remain in nematic state) and dark region corresponds to shined area (*i.e.* molecules transforms from nematic to isotropic state).

## Experimental

### Materials and methods

The analytical grades of ethyl 4-aminobenzoate, sodium nitrite (NaNO_2_), phenol, 1-bromohexane, 1,3-dicyclohexylcarbodiimide (DCC), 4-(*N*,*N*-dimethyl amino)pyridine (DMAP), 4-fluoro aniline, 2-fluoro aniline, 2-nitro aniline, 2-trifluoromethyl aniline, 2,4-dinitro aniline, potassium carbonate (K_2_CO_3_), sodium hydroxide (NaOH), potassium hydroxide (KOH), potassium iodide (KI) were procured from Sigma Aldrich and Spectrochem. For column chromatography silica gel 60-120 and neutral alumina were procured from Thomas baker. Acetone, hexane, ethyl acetate and dichloromethane were dried over phosphorus pentaoxide and calcium hydride, respectively. These solvents were distilled using standard methods when required for the experiment. Thin-layer chromatography (TLC) were performed on aluminium sheet precoated with silica gel-60 F_254_ (Merck). Infrared (IR) spectra were recorded using a PerkinElmer 1000 spectrometer. Proton nuclear magnetic resonance (^1^H NMR) spectra were recorded on a 400 MHz Bruker NMR spectrometer, using CDCl_3_ as a solvent and tetramethyl silane (TMS) as an internal standard.

#### General procedure for the preparation of azobenzene based benzamides

(*E*)-4-((4-(Hexyloxy)phenyl)diazenyl)benzoic acid (0.61 mmol, 1 equiv.) was taken in round bottom flask (RBF) and dissolved in 5 mL of dry dichloromethane (DCM). To this reaction mixture, DMAP (0.061 mmol, 0.1 equiv.) and DCC (0.915 mmol, 1.5 equiv.) was added and stirred for about 30 min. A solution of 4-fluoro aniline (0.61 mmol, 1 equiv.) in dry dichloromethane (5 mL) was added to the reaction mixture and stirred for about 24 h. The completion of the reaction was monitored by using TLC. After completion of the reaction, the reaction mixture was filtered and compound was extracted by chloroform. The extracted organic layer was washed with 1.5 N hydrochloric acid and 1 N sodium hydrogen carbonate followed by a brine solution. The combined organic layer was dried over anhydrous sodium sulphate and concentrated. The crude product was purified by column chromatography using silica gel (60-120 mesh) and hexane : ethyl acetate (7 : 3) as eluent. Finally, the combined column fraction was concentrated to get the pure compound of SG11. A similar procedure was followed to synthesize the compounds SG12, SG13, SG14 and SG15.

#### SG11

(*E*)-4-[(4-Hexyloxyphenyl)diazenyl]-*N*-4-fluoro-phenyl benzamide, orange yellow solid; yield: 45%; IR (KBr Pellet) *γ*_max_ in cm^−1^: 3452, 3015, 2910, 2860, 1718, 1530, 1450, 1420, 1215, 1076, 761. ^1^H NMR (400 MHz, CDCl_3_): *δ* 7.96 (d, *J* = 8.0 Hz, 4H, Ar-**H**), 7.91 (d, *J* = 8.0 Hz, 2H, Ar-**H**), 7.67 (d, *J* = 8.0 Hz, 2H, Ar-**H**), 7.01 (dd, *J* = 8.0, 4.0 Hz, 4H, Ar-**H**), 4.05 (t, *J* = 8.0 Hz, 2H, –OC***H***_2_–), 2.09–1.79 (m, 8H, –(C***H***_2_)_4_–), 0.91 (t, *J* = 12.0 Hz, 3H, –C***H***_3_).

#### SG12

(*E*)-4-[(4-Hexyloxyphenyl)diazenyl]-*N*-2-fluoro-phenyl benzamide, orange yellow solid; yield: 45%; IR (KBr Pellet) *γ*_max_ in cm^−1^: 3425, 3015, 2971, 2843, 1710, 1593, 1425, 1353, 1205, 1022, 772. ^1^H NMR (400 MHz, CDCl_3_): *δ* 7.95 (m, 4H, Ar-**H**), 7.88 (d, *J* = 8.0 Hz, 2H, Ar-**H**), 7.67 (d, *J* = 8.0 Hz, 2H, Ar-**H**), 7.0 (d, *J* = 10.0 Hz, 4H, Ar-**H**), 4.05 (t, *J* = 8.0 Hz, 2H, –OC***H***_2_–), 2.13–1.79 (m, 8H, –(C***H***_2_)_4_–), 0.92 (t, *J* = 8.0 Hz, 3H, –C***H***_3_).

#### SG13

(*E*)-4-(2-(4-(Hexyloxy)phenyl)diazenyl)-*N*-(2-(trifluoromethyl)phenyl)benzamide, orange solid; yield: 35%; IR (KBr Pellet) *γ*_max_ in cm^−1^: 3419, 3020, 2959, 2843, 1719, 1565, 1404, 1385, 1209, 1076, 772. ^1^H NMR (400 MHz, CDCl_3_): *δ* 7.95 (m, 4H, Ar-**H**), 7.86 (d, *J* = 8.0 Hz, 2H, Ar-**H**), 7.65 (d, *J* = 8.0 Hz, 2H, Ar-**H**), 7.00 (d, *J* = 8.0 Hz, 4H, Ar-**H**), 4.05 (t, *J* = 8.0 Hz, 2H, –OC***H***_2_–), 2.13–1.76 (m, 8H, –(C***H***_2_)_4_–), 0.91 (t, *J* = 4.0 Hz, 3H, –C***H***_3_).

#### SG14

(*E*)-4-(2-(4-(Hexyloxy)phenyl)diazenyl)-*N*-(2-nitrophenyl)benzamide, bright yellow solid; yield: 32%; IR (KBr Pellet) *γ*_max_ in cm^−1^: 3420, 3009, 2987, 2883, 1720, 1600, 1414, 1359, 1215, 1071, 758. ^1^H NMR (400 MHz, CDCl_3_): *δ* 7.94 (m, 4H, Ar-**H**), 7.89 (d, *J* = 8.0 Hz, 2H, Ar-**H**), 7.66 (d, *J* = 14.0 Hz, 2H, Ar-**H**), 6.99 (d, *J* = 8.0 Hz, 4H, Ar-**H**), 4.05 (t, *J* = 8.0 Hz, 2H, –OC***H***_2_–), 2.17–1.79 (m, 8H, –(C***H***_2_)_4_–), 0.91 (t, *J* = 4.0 Hz, 3H, –C***H***_3_).

#### SG15

(*E*)-4-(2-(4-(Hexyloxy)phenyl)diazenyl)-*N*-(2,4-(dinitro)phenyl)benzamide, yellow solid; yield: 35%; IR (KBr Pellet) *γ*_max_ in cm^−1^: 3419, 3004, 2992, 2873, 1714, 1586, 1415, 1356, 1215, 1043, 776. ^1^H NMR (400 MHz, CDCl_3_): *δ* 7.94 (m, 4H, Ar-**H**), 7.92 (s, 1H, Ar-**H**), 7.63 (d, *J* = 8.0 Hz, 2H, Ar-**H**), 7.02 (d, *J* = 12.0 Hz, 4H, Ar-**H**), 4.05 (t, *J* = 8.0 Hz, 2H, –OC***H***_2_–), 2.17–1.80 (m, 8H, –(C***H***_2_)_4_–), 0.92 (t, *J* = 8.0 Hz, 3H, –C***H***_3_).

### Mesomorphic studies

The mesophase behaviour of the synthesised azo derivatives was measured using a Linkam hot stage and control temperature under polarized light of an Olympus BX 51 polarizing optical microscope (magnification is 10× and 200 μm). The phase transition temperatures and associated enthalpies were obtained from DSC thermograms which was recorded on a PerkinElmer DSC-7, cooling cycle at the rate of 5 °C min^−1^.

### Photoswitching studies

The photoisomerization of azo derivatives was recorded by using an Ocean Optics HR-2000+ UV-Vis spectrophotometer setup. The experiment is carried out in dark room by using chloroform as a solvent and quartz cuvette of optical length 1 cm. The recorded wavelength range (200 nm to 800 nm) and at fixed concentration of solution is 1.0 × 10^−5^ mol L^−1^. The photostationary state (PSS) of compounds has been investigated by illuminating UV light source (Omni cure series 2000). The intensity of illuminated UV light is 1 mW cm^−2^ (measured by UV meter UV513AB). The UV source equipped with 365 nm wavelength and heat filter to avoid heat radiation from illuminated source to sample. The reverse thermal back relaxation was measured by keeping a sample in a dark at room temperature. The time dependent absorption spectra for all the compounds was plotted as a function of UV irradiation and recovery time during UV illumination and thermal back relaxation, respectively. For reverse thermal isomerization, first-order plots was plotted for all compounds with respect to different intervals of recovery time.

Solid cell was prepared to see their potential efficiency of the given compounds by using previously cleaned ITO coated glass substrate. ITO glass substrate was coated with polyimide solution and rubbed unidirectionally with rayon cloth. Uniform thickness was maintained by spraying glass spacers of 5 μm thickness. The guest–host mixture was prepared by physically mixing with 5% of SG13 in 95% of commercially available liquid crystal E7. Then mixture was filled by using capillary action into the previously prepared cells. The fabrication process is done in the class 1000 clean room and spectral investigation of solid cell was studied using UV-Vis spectrophotometer.

## Conclusions

In summary, new azo derivatives with different lateral/terminal functional groups on phenyl ring attached to the amide moiety were synthesized. The influence of electron withdrawing groups on H-bonding is quite interesting, *para* substituted azo derivative exhibits the intermolecular H-bonding; whereas, intramolecular H-bonding behaviour was noticed for *ortho* substituted compounds. All compounds exhibited liquid crystalline phases except SG13. The photosaturation time of these materials was about 48–60 s and reverse process occurred around 9–13 h in solution. In solid cell, the time required to reach its PSS was around 200 s and around ∼6 h to undergo thermal back relaxation. The changes in thermal back relaxation time was observed due to presence of amide linkage and EWG at *ortho*/*para* position to them in azo derivatives. Surprisingly, EWG substituted at both *ortho* and *para* position to the amide moiety shows slowest thermal back relaxation time compared with mono-substituted. Therefore, electronic effects of substituted groups also make a significant change in optical properties in addition to the H-bonding effect. These types of materials have bright potential in the fabrication of tunable optical data storage devices.

## Conflicts of interest

There are no conflicts to declare.

## Supplementary Material

RA-010-D0RA03024D-s001
