# Supplementary material for: Influence of inter- and intramolecular H-bonding on the mesomorphic and photoswitching behaviour of (E)-4-((4-(hexyloxy)phenyl)diazenyl)-N-phenyl benzamides
Source: RSC Adv. 2020 May 27;10(34):20222–30. doi: 10.1039/d0ra03024d (PMC9054115; doi:10.1039/d0ra03024d)
Supplement: RA-010-D0RA03024D-s001 [file RA-010-D0RA03024D-s001.pdf]

Electronic Supplementary Material (ESI) for RSC Advances.
This journal is © The Royal Society of Chemistry 2020

Supplementary information

Influence of inter- and intramolecular H-bonding on the mesomorphic and photoswitching behaviour of (*E*)-4-((4-(hexyloxy) phenyl) diazenyl)-*N*-phenyl benzamides

Sunil B N,[a,b] Paresh Kumar Behera,[c] Ammathnadu S. Achalkumar,[c] G. Shanker[d] and Gurumurthy Hegde,*[a]

Contents

1. **UV-Vis spectra**
2. **FT-IR spectra of inter- and intramolecular H-bonding**
3. **FT-IR and ^{1}H NMR spectra**

1. UV-Vis spectra: Fig. S1 and S2 shows time dependent absorption spectra during UV illumination and thermal back relaxation of **SG12**, **SG13**, **SG14** and **SG15** with respect to different intervals of time, respectively.

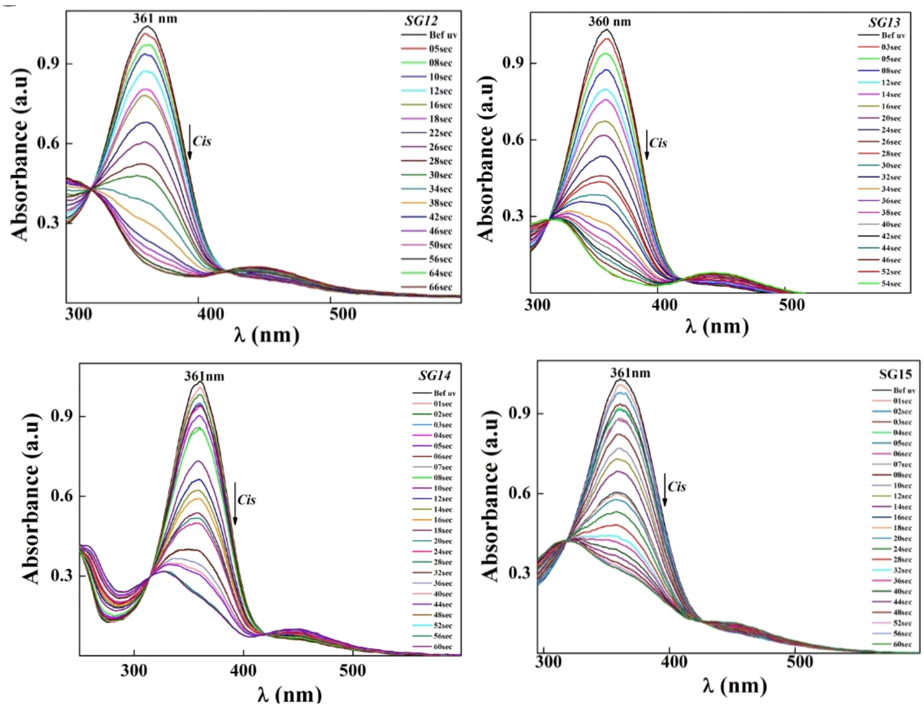

Fig. S1 Time dependent absorption spectra recorded during UV illumination: a) **SG12**, b) **SG13**, c) **SG14** and d) **SG15** with different intervals of UV irradiation time.

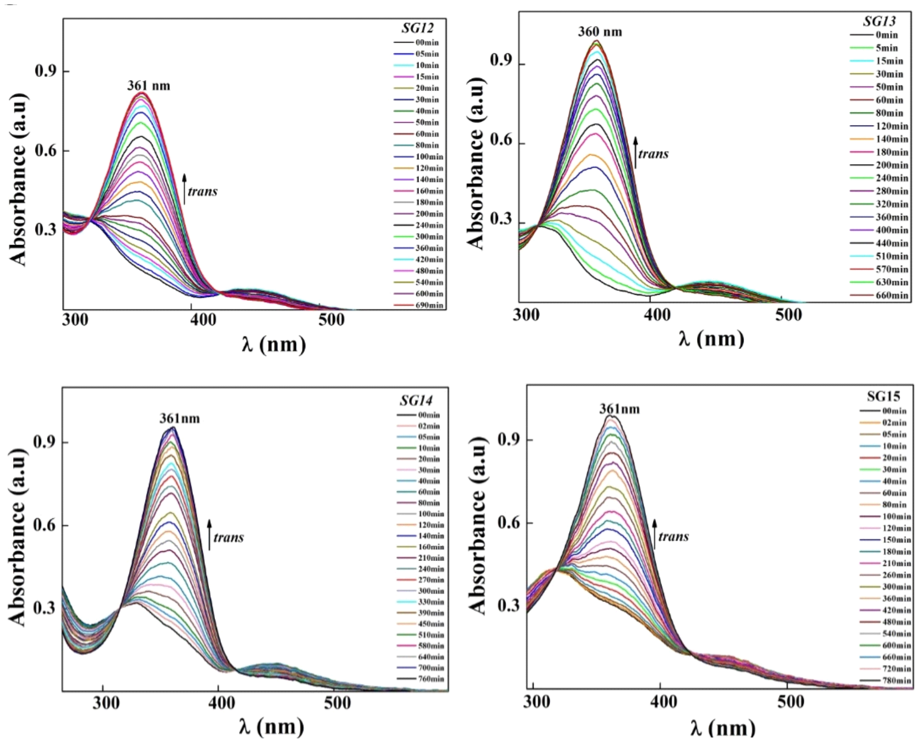

Fig. S2 Time dependent absorption spectra recorded during thermal back relaxation: a) **SG12**, b) **SG13**, c) **SG14** and d) **SG15** with different intervals of recovery time.

2. FT-IR spectra of inter- and intramolecular H-bonding.

Fig.S3 shows illustrative FT-IR spectra corresoponds to inter- and intramolecular H-bonding of compond SG11 and SG14, respectively. Spectra were recorded in solution at different concentration using $CHCl_3$ as a solvent.

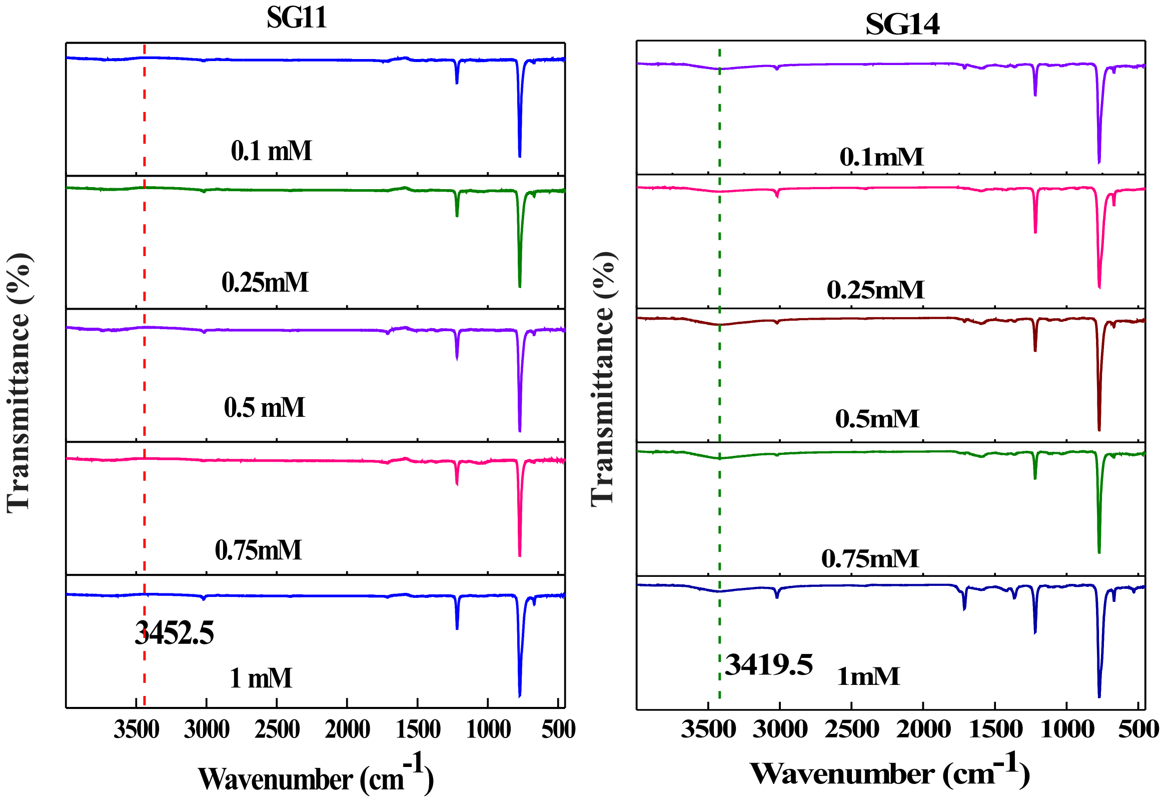

Fig. S3 FT-IR spectra for **SG11** and **SG14** showing inter- and intramolecular H-bonding, respectively. Spectra were recorded in solution at different concentrations using $CHCl_3$ as solvent.

3. FT-IR and ^{1}H NMR spectra

The IR valves of compounds were measured using KBr pellet method in Perkin Elmer FT-IR spectrometer. Fig. S4 represents FT-IR spectra of SG**11, SG12**, **SG13**, **SG14** and **SG15**. ^{1}H NMR spectrum for final compounds spectra were recorded on a 400 MHz Bruker NMR spectrometer, using $CDCl_3$ as a solvent and their respective spectra are given below (Fig. S5- Fig. S9).

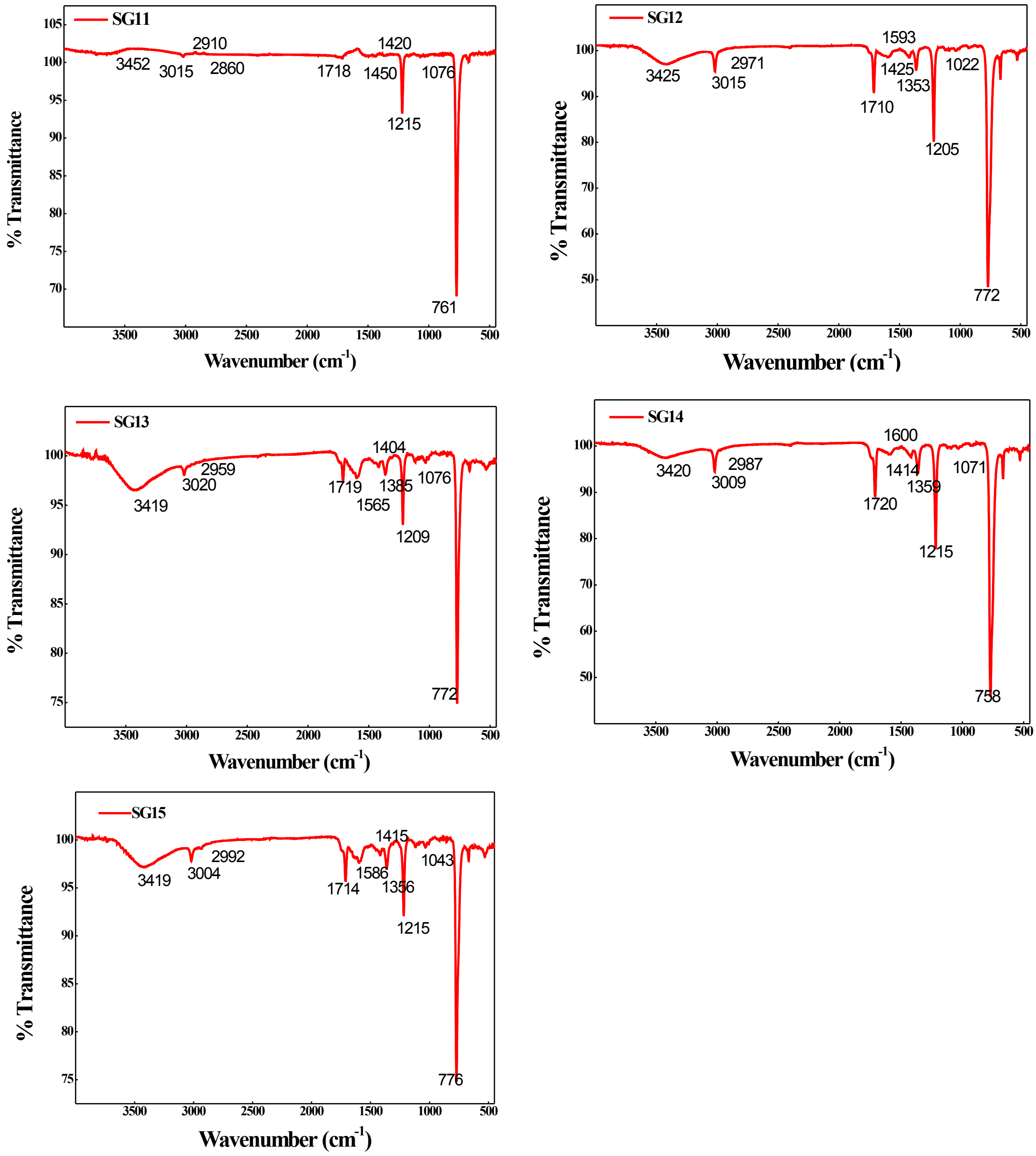

Fig. S4 FT-IR spectrum for final compounds such as **SG11**, **SG12**, **SG13**, **SG14** and **SG15.**

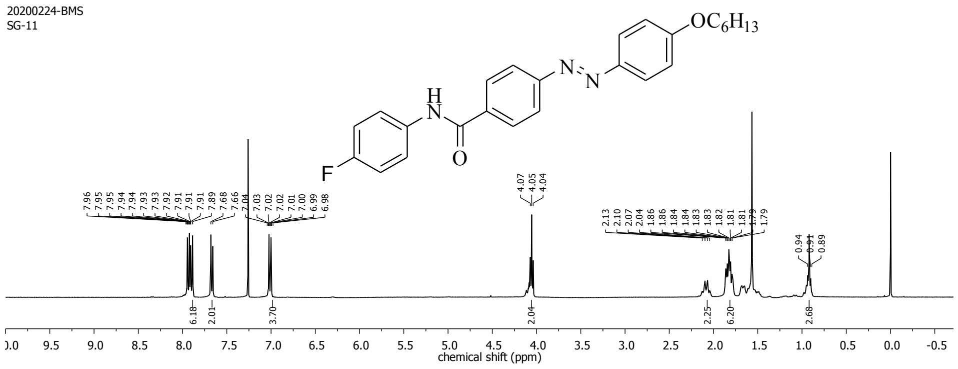

Fig. S5 ^{1}H NMR spectrum for **SG11** (400 MHz, $CDCl_3$).

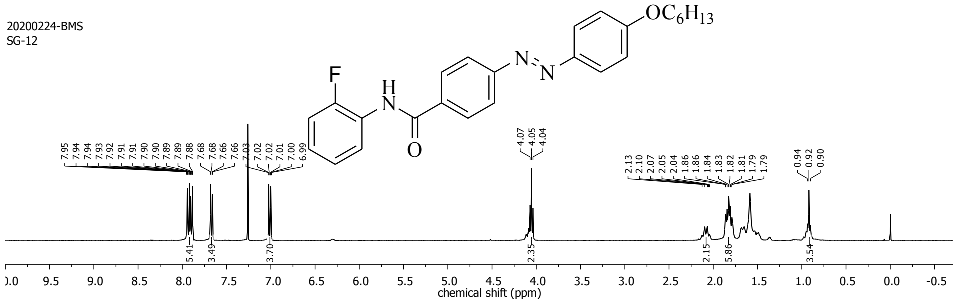

Fig. S6 ^{1}H NMR spectrum for **SG12** (400 MHz, $CDCl_3$).

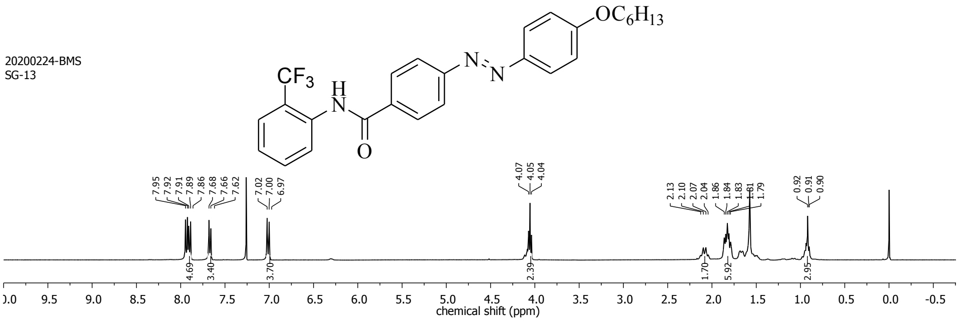

Fig. S7 ^{1}H NMR spectrum for **SG13** (400 MHz, $CDCl_3$).

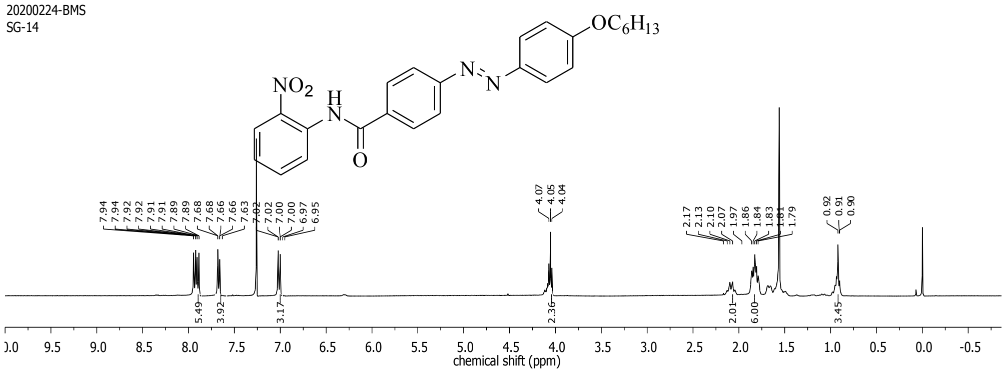

Fig. S8 ^{1}H NMR spectrum for **SG14** (400 MHz, $CDCl_3$).

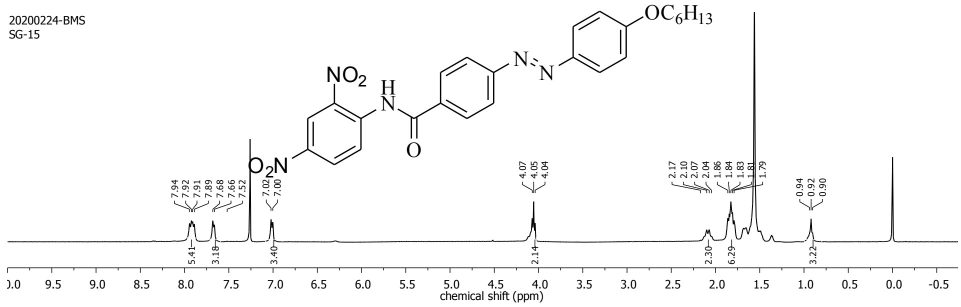

Fig. S9 ^{1}H NMR spectrum for **SG15** (400 MHz, $CDCl_3$).
